# Prevalence and Risk Factors of Burnout Among Female Oncologists From the Middle East and North Africa

**DOI:** 10.3389/fpsyg.2022.845024

**Published:** 2022-03-22

**Authors:** Atlal Abusanad, Assia Bensalem, Emad Shash, Layth Mula-Hussain, Zineb Benbrahim, Sami Khatib, Nafisa Abdelhafiz, Jawaher Ansari, Hoda Jradi, Khaled Alkattan, Abdul Rahman Jazieh

**Affiliations:** ^1^Faculty of Medicine, Medical Oncology, King Abdulaziz University, Jeddah, Saudi Arabia; ^2^Oncology Department, CHU Dr Benbadis, Constantine, Algeria; ^3^Medical Oncology, National Cancer Institute – Cairo University, Cairo, Egypt; ^4^Department of Radiation Oncology, Sultan Qaboos Comprehensive Cancer and Research Center, Muscat, Oman; ^5^Medical Oncology, CHU – University Hospital of Hassan II, Fez, Morocco; ^6^Oncology Department, Private Sector, Amman, Jordan; ^7^Department of Oncology, King Abdulaziz Medical City, Riyadh, Saudi Arabia; ^8^Medical Oncology, Tawam Hospital, Al Ain, United Arab Emirates; ^9^Public Health Faculty, King Saud bin Abdulaziz University for Health Sciences, Riyadh, Saudi Arabia; ^10^Head of Thoracic Surgery at King Faisal Specialist Hospital and Research Center (KFSH-RC), Alfaisal University, Riyadh, Saudi Arabia; ^11^Cincinnati Cancer Advisors, Cincinnati, OH, United States

**Keywords:** burnout, psychology, female oncologists, prevalence, oncology, women in oncology, oncology professionals, MENA

## Abstract

**Background:**

Burnout (BO) is a recognized challenge among the oncology workforce. It affects both genders with a higher frequency among women. This study examined the factors contributing to the development of burnout among female oncologists from the Middle East and North Africa (MENA).

**Methods:**

An online cross-sectional survey was distributed to oncology professionals from different countries in the MENA region. The validated Maslach Burnout Inventory (MBI) of emotional exhaustion (EE), Depersonalization (DE), and Personal Achievement (PA) plus questions about demography/work-related factors and attitudes toward oncology were included. Data were analyzed to measure BO prevalence and related factors.

**Results:**

Between 10 February and 15 March 2020, 545 responses were submitted by female oncologists. The responses pre-dated the COVID-19 pandemic emergence in the region. BO prevalence was 71% among female professionals. Women aged <44 years represented 85% of the cohort. Sixty-two percent were married, 52% with children and one-third practiced a hobby. Two-thirds worked in medical oncology, worked for <10 years and 35% worked in academia. The majority (73%) spent >25% on administrative work daily. Nearly half of the respondents (49%) expressed a recurring thought of quitting oncology and 70% had no burnout support or education. Inability to deliver optimal care was reported as distressing for career development in 82%. Factors significantly influencing the BO risk were identified. Marital status, having children, academia and years in practice did not impact the risk of BO among female oncologists from MENA.

**Conclusion:**

Female oncologists from the Middle East and North Africa (MENA) were found to have a high prevalence of BO. In this cohort, the majority of women oncology workers were young and in their early to mid-career stages. Burnout was linked to being younger, practicing in North African nations, having a heavy administrative load, and having persistent thoughts of quitting. Practicing a hobby and engaging in oncology communication, on the other hand, reduced the chance of BO. Burnout support and education, specifically for oncology women, is required.

## Introduction

The term burnout (BO) in the workplace was first used in the scientific community in the 1970s, describing a psychological state characterized by a lack of motivation, emotional exhaustion, and cynicism in relation to the professional environment (Freudenberger, 1974, 1975). Methods to identify and measure BO have been evolving since then. Maslach led the clinical BO research by creating a validated measurement scale, commonly known as Maslach Burnout Inventory (MBI) (Maslach and Jackson, 1981). Three dimensions typify BO syndrome: Emotional Exhaustion (EE), Depersonalization (DP) and low Personal Accomplishment (PA). Burnout can impact everyone, regardless of career, and is influenced by both the work environment and the nature of the work itself. Several studies have revealed that public service jobs are associated with high levels of burnout. Several studies, for example, have found evidence of burnout among teachers (Pishghadam et al., 2013, 2022; Saboori and Pishghadam, 2016). Likewise, studies worldwide and regionally reported a significant level of BO among health care professionals (HCP) (Dyrbye et al., 2018). A large systematic review of 182 studies that included 109,628 individuals from 45 countries showed that some studies reported BO prevalence as high as 80.5% (Rotenstein et al., 2018). Similarly, a systematic regional review from the Arab world reported a wide range of BO between HCP and subscales; high Emotional Exhaustion (20.0–81.0%), high Depersonalization (9.2–80.0%), and low Personal Accomplishment (13.3–85.8%). Female gender, nationality, service duration, working hours, and shift patterns were all significantly associated with BO (Elbarazi et al., 2017). Specific medical subspecialties workers may manifest BO more than others. A recent large study from the Middle East and North Africa (MENA) evaluated BO among oncologists of both genders showed that being an early-career physician, being from north African countries and having the thought of quitting oncology are associated with increased likelihood to report BO. Likewise, a high level of BO was reported among oncology professionals from different continents (Abusanad et al., 2021).

The female gender has been described as a risk factor for developing burnout, attributed to the female gender’s psychological and physiological nature, and societal factors (Artz et al., 2021). As per the BOMENA study, women made up nearly 50% of the oncology workforce across the MENA region where the prevalence of BO for participants of both genders was estimated at 68%, collectively (Abusanad et al., 2021). However, Female oncologists reported a higher non-significant numerical BO prevalence in the same study. Although several researches addressed the female oncology nurses BO, there is no similar dedicated assessment of BO among female oncology physicians exclusively (Cañadas-De la Fuente et al., 2018). The current study aimed to assess BO prevalence in the light of the participants’ characteristics and explore the factors influencing its risk among female oncologists from MENA.

Burnout is associated with several negative personal, professional, and organizational consequences, which reflect on the offered services leading to reduced quality of care, compromised patients’ safety and increased costs (Dewa et al., 2017; Han et al., 2019; Tawfik et al., 2019). The importance of female oncologists’ wellbeing cannot be emphasized enough when half of the oncology workforce is made up of women. Their wellbeing is crucial to maintain productivity, quality and progress of the oncology care in the region.

## Methods

### Study Design and Data Collection

We conducted a cross-sectional study utilizing an online questionnaire that was constructed on SurveyMonkey^®^ (an online survey development tool) and a link was generated. Between 10 February and 15 March 2020, the survey was made available online to male and female oncologists of all ages. The responses were anonymous and we maintained the confidentiality of all participants in case they provided any identifiers. Institutional Review Board (IR) approval from Alfaisal University, Riyadh, KSA was granted (IRB-20113). There was no compensation for participation. The study was not funded.

### Study Instrument

The questionnaire is comprised of two parts. The first part is assessing the participant’s socio-demographic and work characteristics. The second part is the Maslach BO Inventory–Human Service Survey (MBI-HSS). Periodic reminders were sent every week to achieve the best response rate. Additional questions about the desire to quit, the best and worst aspects of being an oncology professional and the availability of BO support were included. The questionnaire was tested for clarity and feasibility for the target population in an initial pilot study (*N* = 24).

### Scoring the Instrument

The MBI burnout scale includes 3 independent dimensions, Emotional Exhaustion (EE) or depressive anxiety symptoms, Depersonalization (DP) or loss of empathy, and reduction of Personal Accomplishment (PA). Each item is rated on a six-point Likert scale ranging from 0 (never) to 6 (daily). The sum of each MBI scale was computed and the obtained scores were classified according to the previously established recommendations (Maslach and Jackson, 1981). High-level EE was established for scores >30, moderate level of EE for scores ranging from 18 to 29, and low-level of EE for scores ≤17. DP scores ≥12 were considered high-level BO, those ranging from 6 to 11 were considered moderate, and those ≤5 were considered low. PA scores ≤ 33 were considered high-level BO, those ranging from 34 to 39 were considered moderate, and those ≥ 40 were considered low-level BO. A high level of total BO corresponded to high scores for the first two sections (EE and DP) and low scores for the PA section. “High” BO, EE, and DP are expressed as BO, EE, and DP throughout the manuscript to minimize misperception.

### Statistical Analysis

Data of female participants only were extracted and analyzed using statistical STATA statistical software version 14 (College Station, TX, United States). Frequency counts and percentages were calculated for categorical variables, and means and standard deviations (SD) for continuous variables. Pearson’s chi-squared and Fischer’s exact tests (for small counts) were used to assess the independence of various sample characteristics by level of BO. To determine the factors associated with BO among this sample, the unadjusted and adjusted odds ratios (U-OR and Ad-OR) and their corresponding 95% confidence intervals (95% CI) were calculated using logistic regression analysis. Participants with missing data on characteristics considered for the model were excluded from the analysis. The multicollinearity of variables was assessed using collinearity indices and the fit of the multivariate model was assessed using the Hosmer and Lemeshow’s goodness-of-fit test. Statistical significance for all analyses was set at a p-value of less than 0.05.

## Results

A total of 1,054 responses were submitted by oncology physicians of both genders where 545 responses from female oncologists were eligible for analysis. The responses pre-dated the COVID-19 pandemic emergence in the region. The study is a multinational collaboration which was distributed across the majority of middle eastern and north African countries as follows: Algeria, Morocco, Tunisia, Egypt, Saudi Arabia, United Arab Emirates, Bahrain, Qatar, Kuwait, Yemen, Oman, Jordan, Palestinian territory and Iraq. Supplementary Table 1 shows female participants from each corresponding country. Women aged ≤44 years represented 85% of the cohort. Sixty-two percent were married, 52% with children and 33% practiced a hobby. Two-thirds worked in medical oncology, worked for <10 years and 35% worked in academia. The majority (73%) spent >25% on administrative work daily. Nearly half of the respondents (48%) expressed a recurring thought of quitting oncology and 70% had no burnout support or education. Inability to deliver optimal care was reported as a distressing challenge for career development in 82% (Table 1). Burnout prevalence was reported at 71%, EE, DP and low PA were reported at 72%, 82% and 54%, respectively, and several factors were identified to influence the risk of BO (Table 2). Marital status, having children, the number of years in practice, the number of patients seen per week, being in academia and using a paper-filing system did not influence the risk of BO.

**TABLE 1:** Characteristics of participating female oncology physicians.

Demographics	Total N (%)
**Age**	
≤34	275 (50)
35–44	188 (35)
45–54	64 (12)
≥55	18 (3)
**Marital status**	
Married	336 (62)
Separated/Widowed	17 (3)
Single	191 (35)
**Have children**	
Yes	284 (52)
No	261 (48)
**Practice hobby**	
Yes	179 (33)
No	365 (67)
**Work-related variable**	**Total N (%)**
**Sub-specialty**	
Medical oncology	384 (70)
Radiation oncology	80 (15)
Surgical oncology	71 (13)
Other	10 (2)
**Years in practice (N = 1,016)**	
In training/fellow	71 (13)
0–4	147 (27)
5–9	167 (31)
10–19	120 (22)
≥20	39 (7)
**Practice setting (N = 1,015)**	
Academic	191 (35)
Private	72 (13)
Government	316 (52)
**Patient load/week**	
<20	66 (12)
20–30	103 (19)
30–40	97 (17)
40–50	77 (14)
>50	202 (37)
**Administrative work/day**	
<25%	255 (28)
25–50%	244 (45)
>50%	146 (27)
**Medical record system**	
Paper-based	283 (52)
Electronic-based	71 (13)
Mixed	191 (35)
**Attitude toward oncology**	
**More demanding than other specialties**	
Yes	517 (95)
No	28 (5)
**Thought about quitting oncology**	
Always	45 (8)
Sometimes	219 (40)
Rarely	125 (23)
Never	156 (29)
**Best in oncology is science**	
Yes	460 (84)
No	85 (16)
**Best in oncology is money it earns**	
Yes	42 (8)
No	503 (92)
**Enjoying oncology interpersonal relationships and communication**	
Yes	304 (56)
No	241 (44)
**Best in oncology is administrative work**	
Yes	35 (6)
No	510 (94)
**Appreciating oncology life-work balance**	
Yes	118 (22)
No	427 (78)
**Burnout support/education/workshop**	
Yes	162 (30)
No	383 (70)

**TABLE 2:** Multivariate logistic regression analysis for factors affecting the risk of developing burnout in female oncology physicians.

Factors	Ad-OR (CI 95%)	P-value
**Factors associated with increased risk of burnout**		
Age < 44 years	2.26 (1.22–4.19)	0.010
North Africa	2.43 (1.35–4.38)	0.003
Admin work > 50%	1.75 (1.07–2.86)	0.026
Always thinking about quitting	11.91 (3.37–42.05)	<0.001
Sometimes thinking about quitting	5.61 (3.28–9.62)	<0.001
**Factors associated with decreased risk of burnout**		
Practicing hobby	0.60 (0.02–1.52)	0.042
Enjoying inter-personal and professional communication of oncology	0.42 (0.26–0.68)	<0.001

## Discussion

This study investigated burnout prevalence and contributing factors in female oncologists from MENA. It reported a high burnout rate reaching 71% among participants. An interplay of personal, professional and organizational factors can explain the increased burnout prevalence. Female oncologists under the age of 44 were more likely to experience burnout. This can be linked to early-career professionals’ still-evolving personal and professional experience, as well as a lack of support and/or mentoring (Banerjee et al., 2017). Our observation that the majority of female oncologists are young to middle-aged with high burnout supports this finding, as does the fact that there is a growing interest in oncology as a subspecialty among female medical trainees. Nonetheless, this was not matched by the establishment of physician wellbeing and burnout education and support programs. Two-thirds of the participants reported no burnout support or education. Female oncologists practicing in North African countries were twice as likely as women in Middle Eastern countries to experience burnout. Several aspects may explain the association between developing BO and practice location; including the locally applied healthcare system, oncology program staffing ratios, funding, bureaucratic and administrative duties, changing disease pattern, age distribution, and population health state. Being involved with administrative work for more than half of a working day increased the probability of developing burnout. Administrative duties have now become an aspect of patient care on a daily basis. Clinicians and oncologists, in particular, seek professional fulfillment through the ability to connect with and assist patients (Grunfeld et al., 2005; Portoghese et al., 2014). Burdening oncologists with administrative work may prevent them from experiencing professional fulfillment since it reduces the time available to engage directly with patients. Furthermore, the self-perception of being unable to provide optimal care as a result of decreased patient contact time may lead to the development of BO. In this cohort, the inability to provide optimal care was frequently reported (80%).

Female oncologists who responded affirmatively to the desire of quitting oncology demonstrated an increased likelihood to develop burnout regardless of the temporal relationship between being burned out and their willingness to quit oncology. The odd of developing burnout was increased by 12-folds when the thought was “always” recurring. The recurring thought of quitting oncology was explored as a potential screening question in a previous study (Abusanad et al., 2021). The desire of quitting can be explained either by career-choice regret that potentially resulted in burnout or it can be described as a consequence of burnout whereas causality cannot be ascertained.

Although motherhood has been reported as a barrier to advancing career and to achieving life-work balance (Schueller-Weidekamm and Kautzky-Willer, 2012), the association between having children and burnout was not demonstrated in our cohort where nearly half of the participants have children. Likewise, the marital status did not influence the risk of burnout. The majority of studies that described having children as a risk factor for burnout were from western societies (McMurray et al., 2000). The different cultural background and the extended family support in the MENA region could have overcome the shortcomings of being a working mother and its undesirable consequences on life-work balance, thereafter burnout.

Thirty-five percent of female oncologists in our survey worked in academia. This finding may reflect the need to expand women recruitment into academic medicine generally and academic oncology specifically in the region alongside implementing burnout support and education. Contrasting other studies (del Carmen et al., 2019; Merfeld et al., 2021), being in academic oncology did not affect the risk of developing burnout in this cohort possibly due to the small number of women in academic oncology, which was insufficient to demonstrate its association with the risk of burnout.

On the other hand, we identified factors that were associated with decreased risk of developing burnout among female oncologists; enjoying oncology inter-personal relationship and communication and practicing a hobby. Work satisfaction is linked to effective communication (Moore et al., 2013) and the oncology specialty is rich in deep inter-personal relationships and communication. Physicians who appreciate and enjoy these aspects may experience better job satisfaction, which in turn inversely correlates with burnout (Williams et al., 2007; Shanafelt et al., 2014).

Besides the quantitative findings of the survey, individual responses from some participants offered deeper insights into the challenges facing women oncologists in their careers. Almost eighty percent of the female oncologists perceived their inability to provide optimal care as a distressing challenge to their careers followed by the perception of being underappreciated and missing career advancement opportunities which was observed previously in other studies (Chesak et al., 2020). Existing burnout literature proposed several solutions to mitigate and manage professionals burnout on different aspects including personal, professional and organizational levels in general (Olson et al., 2019). However, there is a need to address each community, gender, and career-level in a targeted approach to maximize benefits. In our study, burnout solutions were listed and the majority expressed the need for more time off as a burnout-reducing measure, followed by counseling and education on how to deal with burnout and limiting the time spent on administrative work.

This study is the first in the region to look at burnout among female oncologists, estimate their presence in academic oncology and provide an insight into the challenges they encounter. It also examined how oncology communication and interpersonal relationships, as well as the desire of quitting influenced the risk of developing burnout.

Selection bias is a potential limitation to this study due to differences between those who participated and those who did not in the survey. The number of participants varied between countries, with the highest from North African countries, which could have resulted in a reflection of local trends. Health care models, personal and cultural differences are considerable factors in BO assessment, where region-focused research is worth considering. Focusing on female oncologists burnout and associated factors exclusively distinguished this study and provided an understanding that would help gender-specific intervention to manage burnout.

## Conclusion

Overall, the prevalence of BO among women in oncology from the MENA region is significant. This risk can be reduced by modifying personal and organizational factors. Women oncologists in the region are young and in their early to mid-career stages, which is critical for establishing career paths and enhancing productivity if the right conditions are met. Advocating for physicians’ wellbeing and implementing systematic burnout education and support programs are essential.

## Data Availability Statement

The original contributions presented in the study are included in the article/Supplementary Material, further inquiries can be directed to the corresponding author.

## Ethics Statement

The studies involving human participants were reviewed and approved by the Institutional Review Board of the Alfaisal University, Riyadh (IRB-20113). Written informed consent from the patients/participants or patients/participants legal guardian/next of kin was not required to participate in this study in accordance with the national legislation and the institutional requirements.

## Author Contributions

AA: concept and design and drafting of the manuscript. AA and HJ: statistical analysis. AA and AJ: administrative, technical, or material support and supervision. All co-authors had full access to all of the data in the study and take responsibility for the integrity of the data and the accuracy of the data analysis, and approved the submitted manuscript. All authors: survey distribution, interpretation of data, and critical revision of the manuscript.

## Conflict of Interest

The authors declare that the research was conducted in the absence of any commercial or financial relationships that could be construed as a potential conflict of interest.

## Publisher’s Note

All claims expressed in this article are solely those of the authors and do not necessarily represent those of their affiliated organizations, or those of the publisher, the editors and the reviewers. Any product that may be evaluated in this article, or claim that may be made by its manufacturer, is not guaranteed or endorsed by the publisher.
