# Supplementary material for: Prevalence and Risk Factors of Burnout Among Female Oncologists From the Middle East and North Africa
Source: Front Psychol. 2022 Mar 22;13:845024. doi: 10.3389/fpsyg.2022.845024 (PMC8980775; doi:10.3389/fpsyg.2022.845024)
Supplement: Supplementary file 1 [file Table_1.DOCX]

| Country | number | Percentage |
| --- | --- | --- |
| Algeria | 262 | 48.07% |
| Egypt | 93 | 17.06% |
| Morocco | 66 | 12.11% |
| Iraq | 35 | 6.42% |
| Saudi Arabia | 32 | 5.87% |
| Sudan | 24 | 4.40% |
| Jordan | 8 | 1.47% |
| Syria | 6 | 1.10% |
| Kuwait | 5 | 0.92% |
| Lebanon | 5 | 0.92% |
| UAE | 3 | 0.55% |
| Tunisia | 3 | 0.55% |
| Bahrain | 2 | 0.37% |
| Yemen | 1 | 0.18% |
| Total | 545 | 100.00% |

Supplementary table 1: female oncologists participants ( number and percentage) form corresponding country
